# Differential tissular distribution of *Litomosoides sigmodontis* microfilariae between microfilaremic and amicrofilaremic mice following experimental infection

**DOI:** 10.1051/parasite/2012194351

**Published:** 2012-11-15

**Authors:** T. Bouchery, K. Ehrhardt, E. Lefoulon, W. Hoffmann, O. Bain, C. Martin

**Affiliations:** 1 UMR 7245 MCAM MNHN CNRS & UMR 7205 OSEB MNHN CNRS, Muséum National d’Histoire Naturelle 61, rue Buffon CP52 75231 Paris Cedex 05 France; 2 Institute for Tropical Medicine, University Hospital of Tuebingen, Wilhelmstr. 27 72074 Tuebingen Germany

**Keywords:** filariasis, *Litomosoides sigmodontis*, microfilariae, amicrofilaremic, real-time PCR, lungs, liver, spleen, filariose, *Litomosoides sigmodontis*, microfilaire, amicrofilarien, PCR en temps réel, poumons, foie, rate

## Abstract

Filariases are caused by onchocercid nematodes that are transmitted by arthropod vectors. More than 180 million people are infected worldwide. Mass drug administration has been set up in many endemic areas to control the parasite burden. Although very successful in limiting microfilarial load, transmission has not been completely interrupted in such areas. A proportion of infected patients with lymphatic filariasis or loiasis are known to be amicrofilaremic, as they do not present microfilariae in their bloodstream despite the presence of adult worms. A mirror status also exists in CBA/Ca mice infected with *Litomosoides sigmodontis*, the well-established model of filariasis. Using this model, the goal of this study was to determine if the kinetics of blood clearance of microfilariae differed between amicrofilaremic CBA/Ca mice and microfilaremic BALB/c mice. For this purpose, a qPCR approach was devised to detect microfilariae in different tissues, after a controlled inoculation of microfilariae. We showed that the rapid clearance of microfilariae from the pleural cavity or from the bloodstream of CBA/Ca mice was associated with a massive accumulation of first stage larvae in the lungs, liver and spleen.

## Abstract (fr)

Les infections filariennes (filarioses lymphatiques, onchocercose, loase) affectent plus de 150 millions d’individus dans les régions tropicales et subtropicales. Des traitements de masse ont été mis en place dans les zones endémiques pour contrôler la transmission. Ces derniers, bien que très efficaces pour limiter la charge parasitaire, ne sont pas suffisants pour interrompre totalement la transmission. En zone endémique, une partie des patients infectés par les filarioses lymphatiques ou par la loase sont dits amicrofilariens, c’est-à-dire qu’ils ne présentent pas de microfilaires circulantes. Ce statut amicrofilarien existe aussi chez les souris CBA/Ca infestées par *Litomosoides sigmodontis*. En utilisant ce modèle murin bien établi de filariose, cette étude avait pour but de déterminer si la cinétique d’élimination des microfilaires était différente entre des souris CBA/Ca amicrofilariennes et des souris BALB/c microfilariennes. Pour ce faire, une approche par PCR quantitative a été mise en place afin d’évaluer la quantité de microfilaires présentes dans différents organes des souris après injection contrôlée des premiers stades larvaires. Nous avons pu montrer que la disparition rapide des microfilaires du sang ou de la cavité pleurale des souris CBA/Ca était associée à une forte accumulation des microfilaires dans le foie, la rate et les poumons.

## Introduction

Filariases are vector-borne disabling diseases, mostly tropical. Depending on the species, the filarial nematodes causing the diseases are located in various host tissues, *e.g.* lymphatic vessels, connective tissues or coelomic cavities (Bain & Babayan, 2003; Bain *et al.*, 1994). There, they mature and release microfilariae, which circulate either in the bloodstream for some species or in the dermis for others (Bain & Babayan, 2003). Microfilariae are a major cause of pathogenesis, and their circulation in the peripheral blood or in the dermis, where they can be ingested by their vector, is essential for transmission (Hoerauf *et al.*, 2005; Bain *et al.*, 1994; Aimard *et al.*, 1993, Bockarie *et al.*, 2009). In endemic areas of filariasis with blood-circulating microfilariae, such as lymphatic filariasis, loiasis and mansonellosis, a fraction of concerned patients is amicrofilaremic (up to 40 %, up to 70 % and not known yet respectively), and represents an dead-end for parasite transmission (Remme *et al.*, 2008; Cooney *et al.*, 2010; Simonsen *et al.*, 2011; Taylor *et al.*, 2010). This difference of susceptibility is thought to be immune-dependant because microfilarial vaccination is able to prevent the circulation of microfilariae (Ziewer *et al.*, 2012). Furthermore, several cytokines have been shown to be correlated to the amicrofilaremic status in human and mouse models. Microfilaremia is higher in infected IL-4 KO, IL-5 KO, and IFNγ-KO mice, and intravenously inoculated microfilariae remain longer in the blood circulation before being cleared (Volkmann *et al.*, 2001; Volkmann *et al.*, 2003; Saeftel *et al.*, 2003). In lymphatic filariasis infected individuals, TGF-βsingle nucleotide polymorphism has been associated with microfilaremic/amicrofilaremic status (Debrah *et al.*, 2011) and latent infection of *Wuchereria bancrofti* has been shown to be associated with an elevated adaptive immune response (Arndts *et al.*, 2012). This amicrofilaremic/microfilaremic profile is elegantly mirrored by mice models of filarial disease.

In the mouse model of filariasis infected with *Litomosoides sigmodontis*, destruction and/or non-production of microfilariae has been demonstrated to occur naturally in some strains of mice (Petit *et al.*, 1992; Pfaff *et al.*, 2000). In CBA/Ca mice, microfilariae are never detected in the bloodstream, though adults are fully developed (Petit *et al.*, 1992). On the contrary, in BALB/c mice, infective larvae fully develop into adults capable of releasing microfilariae that persist for weeks (Petit *et al.*, 1992; Pfaff *et al.*, 2000). Disappearance of inoculated microfilariae from the bloodstream takes days or weeks depending on the resistant or susceptible genetic background (Pfaff *et al.*, 2000). Microfilarial detection in the lungs, the liver and the spleen has been associated with the clearance of microfilariae in various filarial models (Wenk, 1986; Pfaff *et al.*, 2000).

Given the difference in the kinetics of microfilariae clearance according to the host’s genetic background, we hypothesize that microfilariae distribution in organs after microfilariae inoculation could differ between microfilaremic BALB/c and amicrofilaremic CBA/Ca mice. In the present study we have defined a new strategy based on qPCR detection to follow the outcome of microfilariae in amicrofilaremic or microfilaremic mice.

## Materials and Methods

### Mice, Infection Models, Necropsies and Tissue Sampling

*Lsigmodontis* is maintained in the MNHN facilities. Infective third-stage larvae (L3) were recovered by dissection of the mite vector *Ornithonyssus bacoti* as previously described, and inoculated to jirds that develop patency (Diagne *et al.*, 1990; Martin *et al.*, 2000). Microfilariae (Mfs) were isolated as described in (Chandrashekar *et al.*, 1984). Briefly, blood from infected jirds was collected and the microfilariae were purified using a percoll density gradient. After lavage in RPMI, microfilariae were counted and 400,000 were inoculated intravenously into the caudal vein of the mice under 50 μL RPMI. For intrapleural inoculations, mice were injected with 200,000 microfilariae under 10 μL of RPMI under transient isofluoran anesthesia.

Six week old female CBA/Ca and BALB/c mice were purchased from Harlan (France) and maintained in the MNHN animal facilities. Kinetics of infection (microfilariae appearance and disappearance from peripheral blood) was followed over three weeks after microfilariae inoculation. The microfilaremia was determined in 10 μL thick blood films and the number of pleural microfilariae was evaluated on pleural wash smears (10 μL of the first milliliter of pleural wash), both stained with Giemsa. An arbitrary score was set up according to the number of microfilariae per smear: +++ > 50,000, ++ > 10,000, + > 1,000. Mice were sacrificed at 15 minutes, one hour, or four days postintravenous inoculation (p.i.) and one day post-intrapleural inoculation. The experimental procedure was carried out in strict accordance with the 2003/65/CEE European directive for animal experimentation.

At necropsy, peritoneal and pleural cavities were opened, and the mice were frozen at - 80 °C for 15 minutes in order to prevent bleeding when removing the tissues. Each tissue sample was collected with different scalpels or scissors to avoid contamination. Lungs, liver, and spleen were then frozen at - 80 °C until DNA extraction.

In order to verify that microfilariae were present in the blood vessels, perfusion of the lungs was done via the right heart with PBS until the perfusion fluid was transparent. These lungs were then removed and frozen at - 80 °C until DNA extraction. Five to six mice were used for each group and experiments were repeated twice.

### Quantitative Appraisal of Mf Tissue Distribution

Lungs, liver, spleen were removed and homogenized on ice with a potter in a fixed volume of PBS (500 μL). The volume of sample used for DNA extraction was standardized to 50 μL. Genomic DNA was extracted with a QIAamp DNA Mini Kit (QIAGEN, Germany) according to the manufacturer’s protocol and finally eluted in 100 μL of sterile water. The total DNA concentration was estimated by spectrophotometric determination of A260 and the concentration was adjusted to 25 ng/μL. Extractions were highly repeatable, and the dilution was thus very similar for each tissue.

Amongst all available *L. sigmodontis* sequence data from the Nucleotide database (NCBI), the mitochondrial cytochrome oxidase subunit Ι gene (COI), a highly conserved sequence within the spirurida subclass, was chosen to quantify microfilariae because targeting mitochondria enhances the sensitivity of the assay. The number of mitochondria is stable at this stage between individuals (Lemire, 2005) and the gene is present in only one copy. No amplification of COI or hsp60 was detected with mouse DNA only. Oligo Calc (Kibbe, 2007) was used to design primer pairs for real time PCR (Table I).

**Table. I.** Sequences of primers for real-time PCR assay.

Assay	Primer	Sequence (5’-3’)	Sequence number
Ls-Co1	AS	5’-ACTGGCCTGGTCTAATGTAACCG-3’	Designed using
	S	5’-GTTGGGGGTGGTCCTGGTAGTA-3’	GenBank: AJ271615.1
Ls-hsp60	AS	5’-ATCATATCCAAACGCCAACTC-3’	(Pfarr *et al.*, 2006)
	S	5’-TACGTGAGCCAATCATGAA-3’
Mm-GAPDH	AS	5’-ACTCTTCCACCTTCGATGCCG-3’	Designed using
	S	5’-ACCCTGTTGCTGTAGCCGTA-3’	GenBank: NM_008084.2

Amplification with primers matching the mouse’s glyceraldehyde 3-phosphate dehydrogenase gene was used as an internal quality control (Table I) but was not used to standardize microfilariae numbers between samples (the global variation of the number of GAPDH amplicons was inferior to 3 %, and was stable due to the fact that microfilarial gDNA is negligible when compared to mouse gDNA in an organ). These primers are specific to mice and do not lead to amplification with *L. sigmodontis* DNA.

The standards were generated for each tissue using for each point one minced organ (lungs, liver or spleen) to which a known number of microfilariae were added before DNA extraction. The standard curve represented the number of microfilariae per whole organ, ranging from 100 to 100,000 for lung and spleen, and from 500 to 100,000 for liver parasites. These standard curves thus allow an absolute quantification of the number of microfilariae in a whole organ.

A real-time PCR was performed with the DNA Master Plus SYBR Green Kit (Roche Diagnostics, Meylan, France) in a LightCycler (Roche Diagnostics) with an initial incubation of ten minutes at 95 °C, 40 amplification cycles of ten seconds at 95 °C, of five seconds at 60 °C, and of ten seconds at 72 °C, during which the fluorescence data were collected. This program was followed by a step of fusion. The 10 μL reaction mixture contained 1X DNA Master Plus SYBR Green, 4 μM of each primer, and 4 μL of template.

### Statistical Analysis

The choice of statistical tests was based on sample size and on Bartlett’s test when normal distribution of the errors was expected. Data from separate experiments were pooled when possible. The microfilaremia kinetics are represented as mean ± SEM of two pooled (intrapleural inoculation) or four pooled (intravenous inoculation) independent experiments, each carried out with six mice per strain, and was analyzed by twoway analysis of variance with repeated measures. The tissular distribution of microfilariae are expressed as the median ± range of six observations for each tissue, represented as stacked barplot and was analyzed by multiple analysis of variance (MANOVA) after logarithmic normalization of data. Representation and data analysis were performed with R (Ihaka et al.,1996) or GraphPad Prism 5. Significant values are indicated as follows: * p < 0.05; ** p < 0.01; *** p < 0.001 and **** p < 0.0001.

## Results

### Validation of the Microfilariae Quantification Pcr-Based Method

**Fig. 1.**
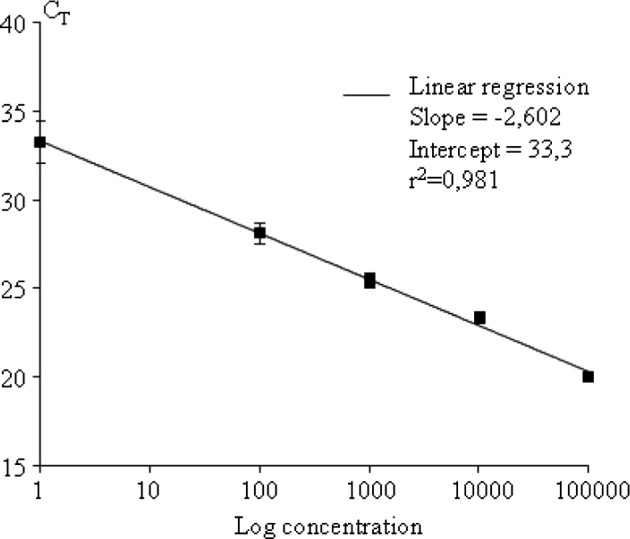
Illustrative curve of COI-PCR assay sensitivity: example of the liver with a range from 100 to 100,000 microfilariae. A plot CT values from four replicates tested on different days against the logarithmic number of microfilariae in whole organ is shown. The results are expressed as mean ± SEM and the variability obtained was similar for the other standard curves generated.

Genomic DNA of microfilariae was amplified by qPCR, and the microfilariae count determined according to a standard curve created for each organ. Each curve, calculated from three independent experiments, was linear over a wide range of microfilariae concentrations [100 to 100,000] for lung and spleen, and [500 to 100,000] for liver parasite per whole organ with correlation coefficients between 0.98 and 0.99 (Fig. 1). Plots of the temperature-dependent dissociation of the SYBR-Green fluorescent marker from COI and GAPDH PCR amplicons showed fluorescence as a single peak, indicating that a single PCR product was generated in each assay. Amplification of the housekeeping gene GAPDH was constant between samples (< 3 % of variations).

### Intrapleurally Inoculated Microfilariae Do Not Reach Peripheral Blood, But Accumulate in the Lungs in Cba/Ca Mice

**Fig. 2.**
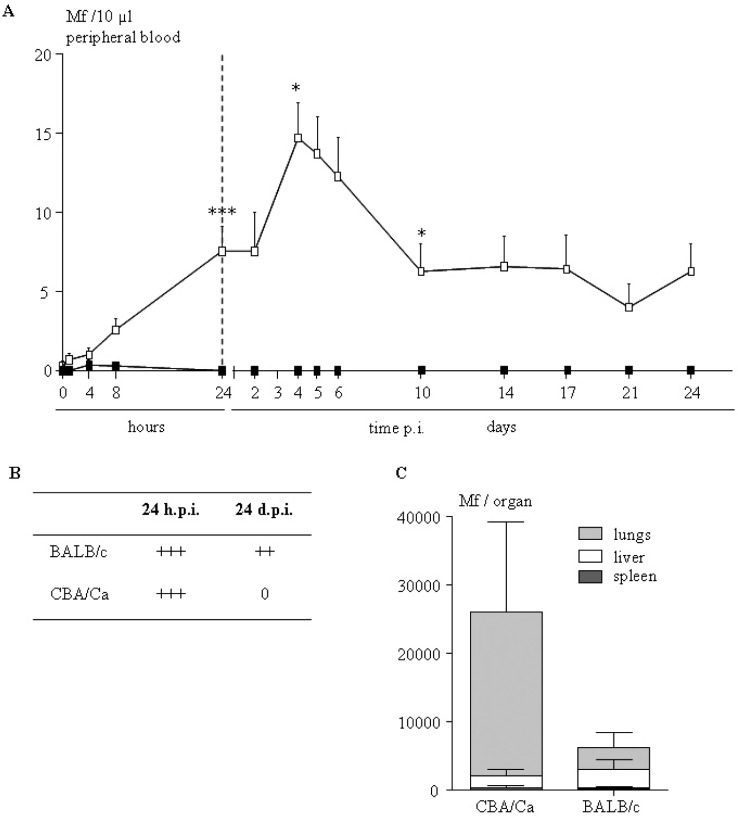
Microfilariae injected in the pleural cavity of CBA/Ca mice did not reach peripheral blood and accumulate in the lungs. A: after inoculation of 250,000 microfilariae into the pleural cavity of BALB/c (white square) and CBA/Ca (black square) microfilaremia was monitored on blood smear over 24 days. Results are presented as mean ± SEM (p < 0.0001); B: microfilarial density in the pleural cavity 24 hours p.i. and 24 days p.i. was evaluated on pleural wash smear. Arbitrary score was set up: +++ > 50,000, ++ > 10,000, + > 1,000; C. parasitic load in the lungs, spleen and liver 24 hours p.i. of CBA/Ca and BALB/c mice was detected by quantitative amplification of COI. Results are expressed as the median + range of six observations for each tissue and represented as a stacked barplot.

The pleural cavity is the site of microfilariae (Mfs) release by adult worms after natural infection, and from there they reach the blood circulation. To analyze the migration of Mfs independently of the adults’ presence and of the acquisition of the immune response they trigger, microfilariae were inoculated directly in the pleural cavity. Appearance of microfilariae in the peripheral blood was monitored over 24 days in microfilaremic BALB/c and amicrofilaremic CBA/Ca mice (Fig. 2A). In microfilaremic BALB/c mice, the kinetics of appearance of microfilariae were slow, taking four days to reach maximal microfilaremia, although it remained low (Fig. 2A). The microfilarial density was then stabilized from ten days until 24 days postinoculation. On the contrary, microfilariae in the blood of CBA/Ca mice were barely detectable on the first day (one mouse positive out of 12) and remained at a null level through the four weeks of experiment (Fig. 2A).

In CBA/Ca mice, microfilariae were thus either not able to exit the pleural cavity or were destroyed before reaching the peripheral blood, *i.e.* in the pleural cavity or during their migration from the cavity to the peripheral blood. Indeed, at one day post inoculation, the number of microfilariae recovered from the pleural cavity was not different between CBA/Ca and BALB/c mice (24 h p.i. 66,500 ± 4,200 and 73,500 ± 2,590 microfilariae in the pleural cavity for CBA/Ca and BALB/c mice respectively, Fig. 2B). However, 24 days post-inoculation, while BALB/c mice were still exhibiting high microfilariae counts in the pleural cavity, none were recovered from CBA/Ca cavity (Fig 2B). No sign of microfilarial destruction or of cell attachment were observed either one day or 24 days post-inoculation in both strains of mice.

As 24 h p.i. is the first discriminating time point between the two studied strains, this time point was used for the analysis of the microfilarial tissue distribution. Although Mfs were mainly detected in the lungs in both CBA/Ca and BALB/c mice, the number of microfilariae was largely and significantly higher in CBA/Ca than in BALB/c mice (about 20,000 and 4,000 microfilariae respectively) (Fig. 2C). Low numbers of microfilariae were also detected in the liver and in the spleen of mice, independently of the strain (Fig. 2C).

### Intravenously Inoculated Microfilariae are Rapidly Cleared From the Peripheral Blood in Cba/Ca Mice

**Fig. 3.**
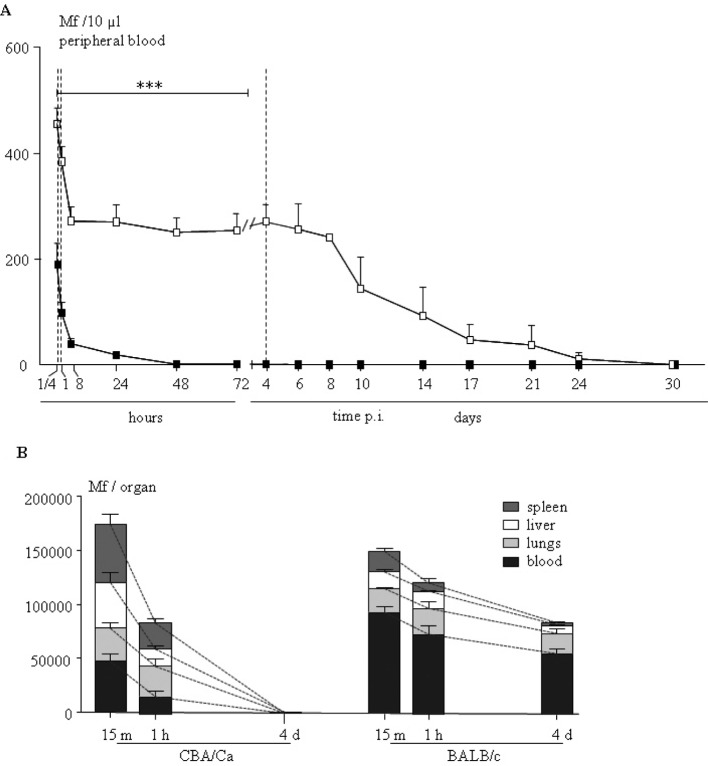
CBA/Ca rapidly cleared intravenously- injected microfilariae from peripheral blood and tissues. A: blood density of microfilariae was monitored on blood smear over 30 days of CBA/Ca (black square) and BALB/c mice (open square). Results are expressed as mean ± SEM (p < 0.0001); B: Parasitic load in the lungs, spleen and liver 15 minutes, one hour and four days p.i. of CBA/Ca and BALB/c mice is detected by quantitative amplification of COI. Results are expressed as the median + range of six observations for tissue and represented as a stacked barplot.

To determine the kinetics of destruction/disappearance of microfilariae in the blood (peripheric or organ blood) independently of the microfilarial migratory pathway from the pleural cavity to the blood, an intravenous inoculation of Mfs was set up. The disappearance of microfilariae in the peripheral blood in CBA/Ca and BALB/c mice was monitored after intravenous inoculation of 400,000 microfilariae (Fig. 3A). In BALB/c mice, elimination of microfilariae from peripheral blood took about four weeks as already described (Hoffmann *et al.*, 2001; Pfaff, 2000) (Fig. 3A). This elimination was in two steps: a first one with a number of microfilariae stable up to heigt days followed by a slow decrease until 30 days post inoculation. On the contrary, the microfilariae in CBA/Ca mice were rapidly cleared from the peripheral blood (Fig. 3A). Indeed, as soon as 15 minutes post-inoculation, the number of circulating microfilariae was significantly lower in CBA/Ca than in BALB/c mice and within four days, it was no longer possible to detect microfilariae in the blood of these amicrofilaremic mice (Fig. 3A).

### Intravenously Inoculated Microfilariae Rapidly Accumulate in Lungs, Liver and Spleen Before A Fast Total Clearance in Cba/Ca Mice

Microfilariae presence in the blood vasculature of the lungs, the spleen and the liver was evaluated by qPCR at 15 minutes, one hour and four days p.i. Microfilariae were present within the blood vessels of the lungs but not inside the lung tissues as confirmed by the absence of microfilarial detection in the lungs after pulmonary perfusion to purge the blood.

The total number of recovered microfilariae was similar in the two strains (about half of the injected number) but the kinetics of microfilariae distribution was different between microfilaremic and amicrofilaremic mice (Fig. 3B): while the number of microfilariae recovered in CBA/Ca mice dropped rapidly and disappeared within four days post-inoculation, the detection of microfilariae in the BALB/c mice was identical throughout the four days of monitoring, even if there was a tendency to decrease from 15 minutes to four days post inoculation (Fig. 3B).

Regarding the detailed tissular distribution (Fig. 3B), more than half of the injected microfilariae were detected in the spleen and the liver of CBA/Ca mice 15 minutes post-inoculation, three times more than in BALB/c mice (p < 0.05). At that time point, most of the microfilariae were still in the blood of BALB/c mice. The number of microfilariae detected in the lungs was equivalent between BALB/c and CBA/Ca mice at this early time point. One hour post-inoculation, the lung-liver-spleen distribution of microfilariae no longer differed between the two strains, stressing that the kinetics of microfilarial clearance were extremely fast in CBA/Ca mice. Then, four days post-inoculation, no more microfilariae were detected in any tissues from CBA/Ca mice, whereas the distribution of microfilariae remained stable in BALB/c mice.

## Discussion and Conclusion

In this article, we describe a highly sensitive and specific method to quantify *L. sigmodontis* microfilariae (Mfs) in mouse tissues by using real-time PCR assays. It defined the difference of microfilariae distribution kinetics between microfilaremic and amicrofilaremic mice. Intrapleuraly and intravenously inoculated Mfs in amicrofilaremic CBA/Ca mice were weakly detected in the peripheral blood, whereas they do accumulate in organs such as lungs, liver and spleen. On the contrary, Mfs in microfilaremic BALB/ c mice are mostly detected in peripheral blood and remain in low quantities in the organs listed above.

Our assay exhibited a reliable performance with a reproducible detection of Mfs from 100 to 100,000 per organ, due to the large copy number of the COI gene, a well-studied gene that can segregate genera of filariae (Ferri *et al.*, 2011). Although this gene is part of the mitochondrial genome, extremely low variations were observed on our standard curves (Fig. 1). This supports the hypothesis that the number of mitochondria is homogenous between first larval stage individuals, as has been demonstrated for *C. elegans* (Lemire, 2005). One limitation of the technique for tissue comparison is the extraction of co-purified inhibitory substances during DNA extraction. In particular, the sensitivity of the assay was lower in the liver than in the spleen and in the lungs. However, as standard curves were generated separately for each tissue analyzed, the bias introduced by natural inhibitors was prevented, thus allowing comparison between organs.

Intrapleurally inoculated Mfs never reached the peripheral bloodstream in amicrofilaremic CBA/Ca mice, unlike in microfilaremic BALB/c mice, as microfilariae accumulate in organ blood circulation. Lung blood vessels have already been described as a reservoir for Mfs during patency for numerous species of hosts and filariae, such as *Brugiamalayi* in jirds, *Loa loa* in mandrills or *L. sigmodontis* in cotton rats (Grove *et al.*, 1979; Pacheco, 1974; Pfaff, 2000). Such an accumulation in the lungs has already been described by Hoffmann *et al.* (Hoffmann, 2001) and has been explained by the high supply in blood of the lungs (Grove *et al.*, 1979; Pacheco, 1974; Pfaff *et al.*, 2000). What is striking in the present study is that the microfilarial pulmonary load is more than three times higher in amicrofilaremic CBA/Ca mice than in microfilaremic BALB/c mice. Indeed, this pattern of accumulation of Mfs in the lungs’ vessels without positive microfilaremia is reminiscent of the development of Tropical Pulmonary Eosinophilia (TPE) in humans. TPE is a rare manifestation of lymphatic filariasis that occurs principally in non-endemic people (Neva *et al.*, 1975; Joe, 1962; Webb *et al.*, 1960). The microfilariae released into the circulation are opsonized with antifilarial antibodies (IgE) and are then cleared in the pulmonary vasculature, causing high levels of eosinophil infiltrate in the lungs and the blood (Vijayan, 2007). The blood leucocyte formula of microfilaremic and amicrofilaremic mice was followed over the course of the infection, but eosinophilia were not observed in either CBA/Ca mice or in BALB/c mice.

No differences were observed in the density of Mfs in the liver and in the spleen between the two strains. However, this does not allow one to entirely exclude a possible role for these organs in the microfilarial clearance, as Mfs can be trapped in the pulmonary circulation just after their exit of the pleural cavity and thus never reach the blood circulation that supplies the spleen and the liver.

In order to determine the kinetics of destruction/disappearance of Mfs in the blood (peripheric or organ blood) independently of the microfilarial migration pathway from the cavity to the blood, an intravenous inoculation of Mfs was set up. Only some of the inoculated Mfs, the proportion of which depends on the genetic background of the mice, are later found circulating in the peripheral blood. Former studies on microfilarial distribution in animal models concluded that non-circulating Mfs were in the blood vessels of deep organs (Grove *et al.*, 1979; Haas et al., 1981; Hoffmann *et al.*, 2001). In humans, several cases of microfilariae detection in spleen, liver, bone marrow, lymph node, bronchial and kidney aspirates have been described (Vij *et al.*, 2011; Vankalakunti *et al.*, 2011; Varghese *et al.*, 1996). The repartition of *L. sigmodontis* microfilariae in BALB/c mice is close to the one described previously for *Sigmodon hispidus*, the natural host of *L. sigmodontis* (Haas et al., 1981; Wenk, 1986; Wenk et al., 1979; Wenk et al., 1982). However, Wenk’s group reported almost no detection of microfilariae in the spleen of the jird, neither after intravenous inoculation nor during patency This difference can be due to technical approaches: in our study, microfilariae are detected by their DNA content, and thus dead or dying Mfs can be detected; furthermore, different susceptibilities of the hosts used for the experiment may influence detection.

In CBA/Ca mice, the kinetics of disappearance of intravenously inoculated Mfs from the peripheral blood is extremely rapid, and is associated with a high Mfs content in organ blood circulation. The higher level of Mfs detection in the spleen and liver of CBA/Ca mice seems to indicate a role of these organs in the destruction of the larvae, as has been postulated by Wenk *et al.* (Wenk, 1986). This is coherent with the reports that the spleen participates in immune responses against Mfs (Duke, 1960; Grove *et al.*, 1979). Indeed, mice inoculated intravenously with Mfs of *Brugia malayi* can present Mfs in the spleen along with a splenic enlargement as soon as two days after infection (Grove *et al.*, 1979). Despite the immune activation of the spleen, only live Mfs were observed in this organ. Mandrills and humans infected with *L. loa* have been reported to present a granulomatous spleen response during patency, and splenectomy largely increases the number of circulating Mfs (Duke, 1960), thus highlighting the importance of the organ for the microfilarial clearance. Previous work on this organ has shown that rat spleen cells are able to adhere to Mfs of *L. sigmodontis in vitro* resulting in damage and death of Mfs by cytotoxicity. This mechanism has been shown to be mediated by an IgE response.

In conclusion, this study defines a novel approach to detect and quantify microfilariae in mouse tissue. The disease outcome depends on a dynamic interaction between the parasite and the host immune system that is tissue and time dependent. Amicrofilaremic mice clear the microfilariae faster by a rapid accumulation of the parasite in the lungs, the spleen and the liver principally. Such results would allow the refinement of immune analyses in a spatiotemporal manner, especially when focusing on the lungs.
